# Identification of Critical States in Complex Biological Systems Using Cell-Specific Causal Network Entropy

**DOI:** 10.34133/research.0852

**Published:** 2025-08-26

**Authors:** Jiayuan Zhong, Ziyi Huang, Jianqiang Qiu, Fei Ling, Pei Chen, Rui Liu

**Affiliations:** ^1^ School of Mathematics, Foshan University, Foshan 528000, China.; ^2^ School of Biology and Biological Engineering, South China University of Technology, Guangzhou 510640, China.; ^3^ School of Mathematics, South China University of Technology, Guangzhou 510640, China.

## Abstract

Abrupt shifts, referred to as critical transitions, are frequently observed in complex biological systems, characterized by marked qualitative changes occurring from one stable state to another through a pre-transitional/critical state. Pinpointing such critical states, along with the signaling molecules, can provide valuable insights into the fundamental mechanisms of intricate biological processes. However, the identification and early warning of the critical state remains a challenge, particularly in model-free cases with high-dimensional single-cell data, where traditional statistical methods often prove inadequate due to the inherent sparsity, noise, and heterogeneity of the data. In this study, we propose a novel quantitative method, cell-specific causal network entropy (CCNE), to infer the specific causal network for each cell and quantify dynamic causal changes, thereby enabling the identification of critical states in complex biological processes at the single-cell level. We validated the accuracy and effectiveness of the proposed approach through numerical simulations and 5 distinct real-world single-cell datasets. Compared to existing methods for detecting critical states, the proposed CCNE exhibits enhanced effectiveness in identifying critical transition signals. Moreover, CCNE score is a computational tool for distinguishing temporal changes in cellular heterogeneity and demonstrates satisfactory performance in clustering cells over time. In addition, the reliability of CCNE is further emphasized through the functional enrichment and pathway analysis of signaling molecules.

## Introduction

**Fig. 1.**
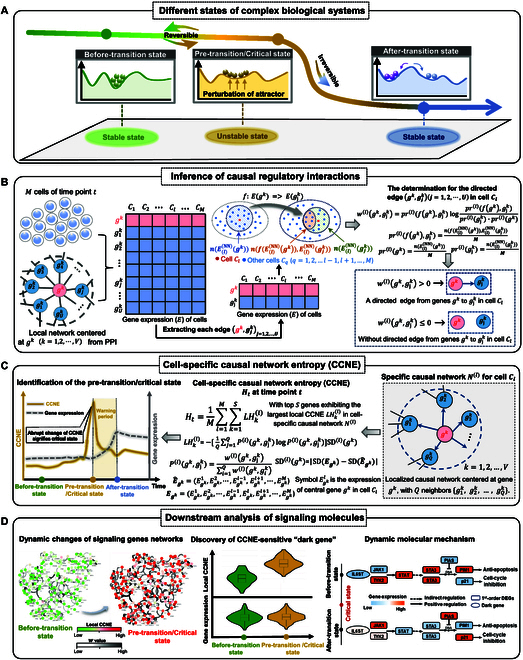
A schematic depiction of the proposed CCNE for identifying pre-transition states in complex biological systems. (A) The complex biological process can be generally classified into 3 distinct states: a stable before-transition state, a highly sensitive and unstable pre-transition state where cell state transition occurs, and another stable after-transition state. (B) The causal relationship and its strength between 2 molecules can be inferred using the continuity scaling law, allowing the construction of cell-specific causal networks for each cell. (C) The local CCNE value is computed for each localized causal network, while the CCNE at each time point is utilized to assess the criticality of the complex biological system process, with a marked increase indicating an approaching pre-transition or critical state. (D) Three main analyses are performed using CCNE: the dynamic changes in signaling gene networks, identification of CCNE-sensitive “dark genes”, and exploration of functional pathways implicated in signaling gene.

The complex biological processes, such as pericyte-to-neuron transition [1], pluripotency-to-hepatocyte transition [2], and epithelial-to-mesenchymal transition [3], involve pre-transition or critical states where marked and qualitative shifts occur. From a dynamics viewpoint, complex biological processes are typically seen as a time-dependent nonlinear dynamical system, characterized by 3 main phases: a before-transition state with stability and resilience, a pre-transition state marked by high sensitivity and instability where cell state transition occurs, and a subsequent stable after-transition state (Fig. 1A) [4]. Identifying the critical state and associated key molecules is pivotal for many biological phenomena, such as cell differentiation, where it is crucial for cellular reprogramming and holds great significance for progress in regenerative medicine [5]. However, the high dimensionality, non-linearity, and dynamic complexity of biological systems make it difficult to detect precursory signs of the pre-transition state, especially when working with inherently sparse, noisy, and heterogeneous single-cell datasets. Although various methods have achieved effective outcomes in single-cell data analysis, particularly in cell clustering, trajectory inference, and cellular heterogeneity [6–8], the task of analyzing molecular causal regulatory relationships and predicting pre-transition states continues to present a great challenge [9,10].

Our recently proposed concept, the dynamic network biomarker (DNB), rooted in nonlinear dynamical systems theory, has been developed to identify critical states and predict key molecules in complex biological processes by focusing on groups of collectively fluctuating genes. The DNB method and its extended versions have been successfully applied to identify pre-disease states for complex diseases [11–13], uncover cell-fate transitions during embryonic development [14], and explore the mechanism of immune checkpoint blockades [15]. However, these approaches mainly aim to detect early warning signals of critical transitions by utilizing the dynamical characteristics of critical states derived from bulk omics data and still continue to face robustness issues in high-noise data, particularly evident in single-cell transcriptomic analysis. Among omics data, single-cell data stand out for their ability to uncover gene associations and offer a wealth of unprecedented information crucial for detecting critical states [16,17]. Causality represents a directional relationship between 2 variables and is a fundamental interaction in complex systems, providing valuable insights to advance our understanding of system dynamics. For instance, causality metrics derived from Takens’ embedding theorem effectively capture the relationship between non-linearly coupled nodes and tackle a key causal inference issue related to non-separability [18]. Combining causality measures with the early-warning signal framework has the potential to enhance the robustness and informativeness of pinpointing critical transitions or system bifurcations [18,19]. Therefore, it is necessary to develop innovative causality network-based methods tailored to single-cell expression data, enabling the identification of pre-transition states in complex biological processes and the discovery of the associated key molecules.

In this study, from the perspective of gene-level causal relations, we introduce a novel quantitative method, cell-specific causal network entropy (CCNE), to infer a distinct causal network for each cell and identify the pre-transition state of complex biological system by analyzing the dynamic changes in molecular causal relationships. Specifically, the cell-specific causal network is constructed based on the continuity scaling law [20,21], a rigorous mathematical framework consistent with the natural interpretation of functional dependency, allowing for the quantification of “continuous causality” and its causal strength between 2 molecules through varying neighbor size (Fig. 1B). Furthermore, the local CCNE is calculated to evaluate the dynamic alterations of causal relationships among molecules within localized causal networks, and the CCNE at each time point serves to quantify the criticality of complex biological process, with a marked increase indicating an impending pre-transition or critical state (Fig. 1C). To demonstrate the reliability and effectiveness of CCNE, we conducted a verification using a numerical simulation and 5 distinct real-world single-cell datasets. Our results show that CCNE is more effective and accurate in pinpointing pre-transition states compared to existing methods, with the predicted pre-transition states aligning well with experimental observations. Furthermore, by converting the sparse single-cell expression matrix into a non-sparse entropy matrix, CCNE can effectively distinguish cellular heterogeneity over time and facilitate analysis of temporal clusters. Besides, we highlight the effectiveness of CCNE signaling biomarkers in downstream functional analyses (Fig. 1D). Therefore, we present an innovative computational method tailored for single-cell data, which provides a new framework to track the dynamics of biological systems in terms of cell-specific causal networks.

## Results

### Performance and resilience of CCNE approach for simulation data

**Fig. 2.**
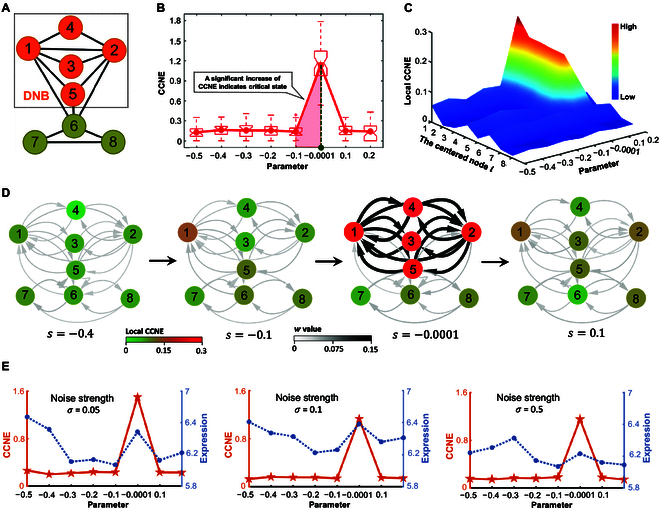
Performance and resilience of CCNE approach for simulation data. (A) An 8-node regulatory network employed as the foundation for generating numerical simulations. (B) The CCNE score exhibits a sharp increase near the bifurcation or tipping point (s=0). (C) The dynamics landscape of local CCNE score is illustrated across different nodes. Remarkably, certain nodes identified as DNBs (nodes 1 to 5) show a marked increase when the system approaches the critical state. (D) The dynamic evolution of the regulatory network reveals a pronounced change in the configuration of DNB subnetworks near the bifurcation point. (E) A comparison between the performance of CCNE and molecular expression reveals that the CCNE method is more robust and effective in detecting critical signals.

To evaluate how well the CCNE approach performs and its ability to withstand noise condition, we utilized an 8-node regulatory network (depicted in Fig. 2A) determined by a set of stochastic differential equations (Eq. S1) to simulate the behavior and evolution of complex biological systems. Such a regulatory network based on Michaelis-Menten form or Hill bifurcation is commonly applied to model how genes regulate activities in biological systems, including transcription and translation [22,23]. The network undergoes a critical transition governed by the parameter s, where s =0 denotes the bifurcation point. Nodes 1 to 5 serve as DNBs or signaling molecules, while the other nodes are classified as non-DNBs. We performed numerical simulations, varying parameter s from −0.5 to 0.2, to illustrate the effectiveness of our CCNE approach in identifying the critical phase of the system near the bifurcation point. More details about the dynamical system are located in Section A of the Supplementary Materials.

As illustrated in Fig. 2B, the CCNE score demonstrates a marked increase near the bifurcation or tipping point (s = 0), signalling an imminent critical state. Conversely, when the system is distant from this point, the CCNE score consistently remains low. Moreover, the dynamics landscape of the local CCNE score in Fig. 2C is depicted to showcase the specific behaviors of each node or molecule. Clearly, when the system is far from the critical state, the local CCNE scores of all nodes remain low and stable, while certain nodes identified as DNBs (nodes 1 to 5) show a notable rise as the system approaches the critical state. Furthermore, the dynamic evolution of the regulatory network is showcased in Fig. 2D, revealing a dramatic change in the configuration of DNB subnetworks near the critical state, which indicates an impending transition in the network state. Besides, we conducted a comparative analysis between CCNE and molecular expression using different noise-perturbed samples to highlight the resilience of the proposed method (Fig. 2E). With increasing noise strength σ, our CCNE method proved to be more robust and effective in detecting critical signal. The numerical experiment illustrates that the proposed SCNE method identifies pre-transition or critical states with reliability and accuracy.

### Identification of the pre-transition state for different biological processes

To illustrate the functionality of the proposed CCNE, we implemented it on 5 distinct single-cell datasets: pericyte-to-neuron transition [24], mouse embryonic fibroblast (MEF)-to-neuron transition [25], mouse hepatoblast cell (MHC)-to-hepatocyte and cholangiocyte cell (HCC) transition [26], epithelial cell deterioration (EPCD) transition [27], and induced pluripotent stem cell (iPSC)-to-mature hepatocyte (MH) transition [28]. The CCNE score specific to each cell was determined using Eq. 5 presented in Materials and Methods. Then, the average CCNE score at a specific time point was employed to detect potential critical signals. For the above datasets, our CCNE approach effectively pinpointed the pre-transition states during biological processes, demonstrating its accuracy and effectiveness. The algorithm’s source code can be freely accessed at https://github.com/zhongjiayuan-fs/CCNE_projects.

**Fig. 3.**
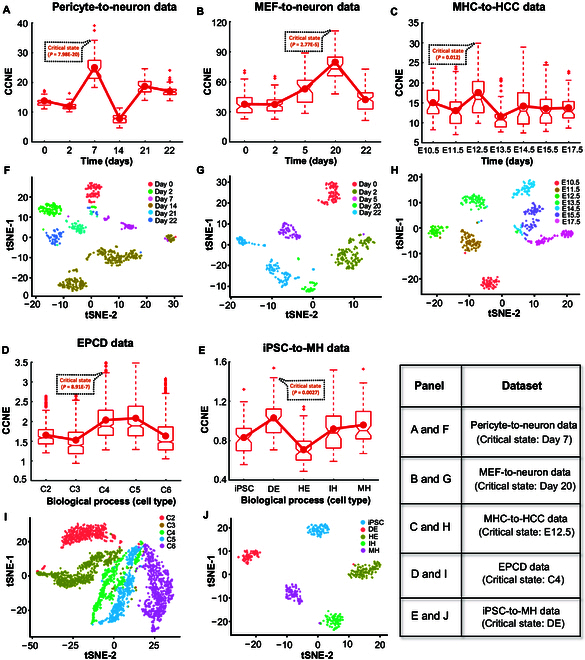
Identification of the pre-transition state for complex biological process. The performance of the CCNE approach for 5 single-cell datasets related to cell-fate transition processes: (A) pericyte-to-neuron data, (B) MEF-to-neuron data, (C) MHC-to-HCC data, (D) EPCD data, and (E) iPSC-to-MH data. Temporal clustering analysis based on the CCNE matrix was conducted for 5 single-cell datasets of different biological processes: (F) pericyte-to-neuron data, (G) MEF-to-neuron data, (H) MHC-to-HCC data, (I) EPCD data, and (J) iPSC-to-MH data.

In pericyte-to-neuron data, Fig. 3A illustrates a notable rise in the average CCNE score on day 7 (*P* = 7.98E−20), indicating an early indication of differentiation into induced neurons [24]. For MEF-to-neuron data, the average CCNE score depicted in Fig. 3B has shown an upward trend from day 5 to day 20 (*P* = 2.77E−5), after which it was observed that mouse embryonic intermediate cells are differentiated into induced neurons [25]. For MHC-to-HCC data, as presented in Fig. 2C, a significant change of average CCNE score is evident at embryonic day 12.5 (E12.5) (P=0.012). This result is consistent with findings from the original experiment that hepatoblasts differentiate into hepatocytes and cholangiocytes after E12.5 [26]. For the non-time-series single-cell dataset of EPCD, the progression of EPCD can be categorized into 6 distinct clusters by constructing a pseudo-temporal trajectory (see Section B of the Supplementary Materials for details). It is seen from Fig. 3D that a marked shift (*P* = 8.91E−7) in the average CCNE score appears at cluster 4 (C4), signaling a critical transition toward deteriorated epithelial cells and uncovering a subset of epithelial cells in the pre-deterioration stage [27]. When applied to iPSC-to-MH data, Fig. 3E shows a significant increase (P=0.0027) in the average CCNE score observed in the definitive endoderm (DE) cell type, followed by the transition of DE cells into hepatic endoderm (HE) cell types [28]. In addition, the median values displayed in the red box plot of the CCNE score in Fig. 3A to E emphasize the robustness of our CCNE in pinpointing critical state. Besides, the proposed CCNE achieves better performance than existing critical state detection methods, including single-cell gene association entropy (SGAE) [29], single-sample landscape entropy (SLE) [30], BioTIP [31], and landscape DNB (LDNB) [32], for identifying pre-transition states during complex biological processes (Table 1). However, from the perspective of gene expression patterns, the dynamic changes and critical states of complex biological processes cannot be characterized as effectively by the CCNE value (Fig. S2). In addition to identification of the pre-transition state, our approach can transform gene expression data into a CCNE matrix, enabling CCNE-based cell clustering analysis. As illustrated in Fig. 3F to J, CCNE-based clustering analysis can effectively group cells from different time points or types utilizing t-distributed stochastic neighbor embedding (t-SNE) [33]. Similarly, the Uniform Manifold Approximation and Projection (UMAP) results distinguish cellular states at various time points as effectively as the t-SNE analysis, indicating that the day-to-day transitions can also be captured by CCNE scores using UMAP-based clustering (Fig. S3).

**Table 1.** Comparison of the performance among different critical state detection methods

Dataset	Pericyte to neuron	MEF to neuron	MHC to HCC	EPCD	iPSC to MH
CCNE	Day 7 (*P* = 7.98E−20)	Day 20 (*P* = 2.77E−5)	E12.5 (*P* = 0.012)	Cluster 4 (*P* =8.91E−7)	DE (*P* = 0.0027)
SGAE	Day 7 (*P* = 6.28E−4)	Day 20 (*P* = 0.0027)	E12.5 (*P* = 0.023)	None	None
SLE	Day 14 (*P* = 0.064)	Day 22 (P = 2.72E−7)	E14.5 (*P* = 0.0039)	Cluster 6 (*P* = 2.18E−5)	MH (*P* = 2.11E−6)
BioTIP	Day 14 (*P* = 7.53E−10)	None	E15.5 (*P* = 0.008)	Cluster 5 (*P* = 1.69E−21)	DE (*P* = 0.0082)
LDNB	Day 14 (*P* = 0.053)	Day 20 (*P* = 1.71E−7)	E13.5 (*P* = 0.013)	Cluster 6 (*P* = 0.024)	IH (*P* = 1.13E−6)

MEF, mouse embryonic fibroblast; MHC, mouse hepatoblast cell; HCC, hepatocyte and cholangiocyte cell; EPCD, epithelial cell deterioration; iPSC, induced pluripotent stem cell; MH, mature hepatocyte; CCNE, cell-specific causal network entropy; SGAE, single-cell gene association entropy; SLE, single-sample landscape entropy; LDNB, landscape dynamic network biomarker

### Evolution of regulatory network and identification of “dark genes” for signaling genes

**Fig. 4.**
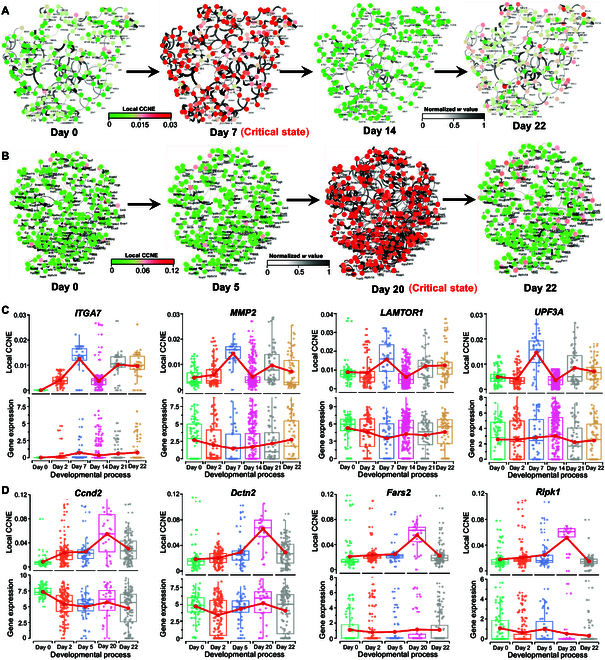
Dynamic evolution of regulatory networks and identification of "dark genes" for signaling genes. Dynamic changes of signaling gene networks are shown for (A) pericyte-to-neuron data and (B) MEF-to-neuron data. The local CCNE score (top) and gene expression value (bottom) of “dark genes” are depicted for (C) pericyte-to-neuron data and (D) MEF-to-neuron data.

At the determined critical state, the top 5% of genes exhibiting the highest local CCNE values were selected as candidate signaling genes for further functional and biological process analysis. Specifically, they were mapped onto the protein–protein interaction (PPI) network, from which the most extensive connected subgraph was extracted for exploring the dynamic changes of the signaling genes network. For pericyte-to-neuron data, a noticeable shift in the network structure on day 7 was observed (Fig. 4A), implying that cell fate begins to transition toward the differentiation into induced neurons after day 7 [24]. When applied to the MEF-to-neuron data, there is a distinct alteration in the network structure on day 20 (Fig. 4B), suggesting an impending critical transition of mouse embryonic intermediate cells differentiating into induced neurons by day 22 [25]. The overall dynamic evolution of the regulatory network for these 2 datasets is illustrated in Fig. S4. Moreover, signaling genes are utilized to discover non-differential genes sensitive to the CCNE score, which may play an important role in biological processes. Despite its effectiveness in discovering new drug targets and biomarkers, differential expression analysis typically fails to consider non-differentially expressed genes (non-DEGs) that are vital to key biological functions. It is evident that such genes are involved in immune cell function [34] and are commonly associated with development pathways [35]. In our study, some genes among DNBs or signaling molecules do not exhibit differential expression at the molecular level but are highly responsive to variations in the CCNE score. Signaling genes are categorized as “dark genes” and meet 2 criteria: (a) they do not differ significantly at the level of gene expression, and (b) they demonstrate a significant distinction between before-transition and pre-transition states based on CCNE score. Specifically, focused on signaling molecules, we compared the dynamic changes of each gene at both the gene expression and CCNE score levels to discover “dark genes”. As depicted in Fig. 4C and D, some “dark genes” identified in the pericyte-to-neuron and MEF-to-neuron data show no significant differences in gene expression, yet there are notable changes in the CCNE score. Additional dark genes for pericyte-to-neuron, MEF-to-neuron, and iPSC-to-MH data can be found in Section F of the Supplementary Materials. Notably, the CCNE score of “dark genes” exhibits marked upward trends, serving as an early indication of the upcoming pre-transition state. This finding also underscores the capability of the CCNE algorithm to accurately forecast impending critical states. In addition, Table 2 illustrates that certain “dark genes” have been demonstrated to play crucial functional roles in the associated biological processes. Moreover, according to Gene Ontology enrichment analysis, the “dark genes” are enriched in pathways functionally associated with cellular proliferation, tissue differentiation, and morphogenesis during embryonic development (Fig. S5). In addition, these “dark genes” are capable of regulating various non-coding RNAs (Fig. S6A and B). Specifically, *ITGA7*, *MMP2*, and *LAMTOR1* are likely to interact with specific microRNAs (miRNAs) that are mainly enriched in embryogenesis-related pathways (Fig. S6C), such as the Wnt signaling pathway [36], the JAK-STAT signaling pathway [37], and the FoxO signaling pathway [38], suggesting their potential roles as regulatory factors during embryonic development. Similarly, “dark genes” including *Ccnd2, Dctn2, Fars2, and Ripk1* may contribute to embryonic development through the regulation of miRNAs associated with developmental pathways (Fig. S6D).

**Table 2.** The information of important “dark genes”

Gene	Location	Relation with embryonic development	PMID
*ITGA7*	Plasma membrane	The down-regulation of *ITGA7* could potentially hinder the process of cell differentiation during neural tube development.	33491544
*MMP2*	Nucleus	*MMP2* treatment of iPSCs enhanced their proliferation and differentiation into neural stem cells.	29137974
*LAMTOR1*	Lysosome	*LAMTOR1* regulates embryonic stem cell differentiation by GTPases Rag activation, promoting the exit from pluripotency.	28935770
*UPF3A*	Cytosol	*UPF3A* inhibits nonsense-mediated decay to promote the expression of genes involved in neuronal differentiation.	27837180
*Ccnd2*	Cytosol	*Ccnd2* can control cell fate decisions in human pluripotent stem cells by recruiting transcriptional corepressors.	26883361
*Dctn2*	Cytosol	*Dctn2 i*s essential for normal neuronal function and may play a role in neuronal differentiation and neuroprotection.	31115483
*Fars2*	Mitochondrion	*Fars2* deficiency affects neuronal development and enhances neuronal apoptosis by impairing mitochondrial function.	35794642
*Ripk1*	Cytosol	*Ripk1* is a key effector in necroptosis and can regulate both apoptosis and necroptosis during embryonic development.	30867408

### Uncovering potential regulatory mechanisms in cell development and differentiation

**Fig. 5.**
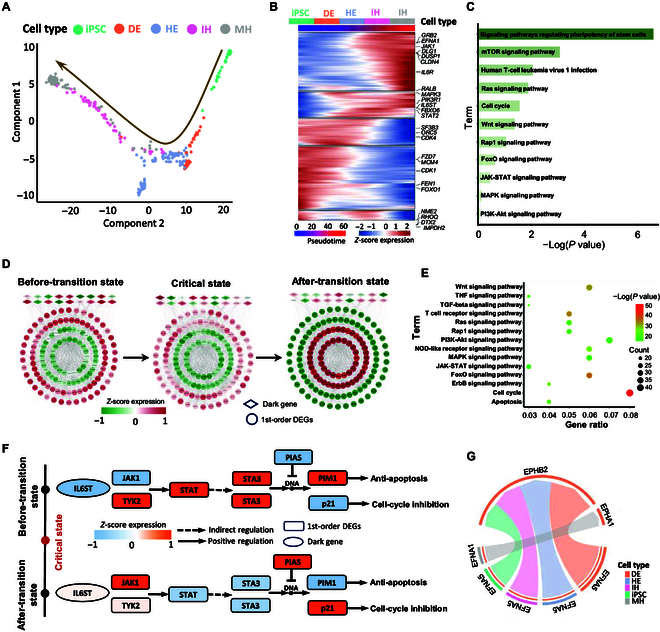
The potential regulatory mechanisms during iPSC cell development and differentiation. (A) Inferred potential differentiation trajectory from iPSC cells to DE, HE, IH, and MH cells. (B) The heatmap for the temporal sequence of gene expression events during iPSC cell development and differentiation. (C) The primary enriched KEGG pathways for dark genes. (D) Dynamic evolution of the PPI subnetwork of dark genes and their first-order DEG neighbors for the iPSC differentiation process. (E) The KEGG pathway enrichment analysis for the first-order DEG neighbors. (F) The potential regulatory mechanism is uncovered by the functional analysis of dark genes and their first-order DEG neighbors. (G) The chord plot shows the interactions among cell subpopulations along the iPSC differentiation trajectory via the EPHA signaling pathway.

Monocle analysis was performed on single cells from various cell types to infer the lineage relationships during iPSC development (Fig. 5A). Subsequently, by heatmap visualization of Monocle-derived gene expression, we uncovered the temporal sequence of gene expression events during differentiation (Fig. 5B). These temporally ordered changes in gene expression of signaling molecules and first-order neighbor may regulate iPSC differentiation, revealing the lineage progression from iPSC cells to DE, HE, immature hepatocyte (IH), and MH cells. In addition, Fig. 5C shows that the primary enriched pathways for dark genes or a specific group of signaling molecules are closely related to pluripotent stem cell development and differentiation. To further explore the regulatory mechanisms of iPSC differentiation, we analyzed the dynamic changes in the subnetwork formed by dark genes and their first-order DEG neighbors within the PPI network (Fig. 5D), which offers a detailed insight into the interactions and relationships influencing iPSC cell differentiation. In our research, the first-order DEG neighbors are identified based on the following 2 criteria: (a) they are directly connected to dark genes in the PPI network, and (b) they are DEGs, exhibiting statistically significant expression changes before and after the identified critical transition state. Before and after the critical state, there was a substantial shift in gene expression within the subnetwork, marked by a marked increase from low to high levels. Additionally, Kyoto Encyclopedia of Genes and Genomes (KEGG) pathway analysis revealed that first-order DEG neighbors are primarily enriched in pathways related to cell development and differentiation (Fig. 5E). For example, the cell cycle pathway was crucial in regulating cell proliferation and differentiation [39], while the mitogen-activated protein kinase signaling pathway influenced various cellular processes, including differentiation and development [40]. Furthermore, the JAK-STAT signaling pathway was critical in regulating iPSC reprogramming, maintaining pluripotency, and guiding differentiation [41]. Specifically, we found that the long-range signaling axis involving *IL6ST* and *JAK* may exhibit dynamic spatiotemporal changes during iPSC lineage progression. As illustrated in Fig. 5F, *IL6ST* (a dark gene), as an upstream growth factor in the JAK-STAT pathway, activates JAK signaling after crossing the critical state, indicating that signaling begins during this period. Concurrently, the expression of downstream *JAK1* (first-order DEG neighbor) was up-regulated, enhancing the cell’s response to factors like *IL-6* and promoting downstream signal transduction. Following *IL6ST* activation of various regulatory and downstream receptors, both *STAT3* activation and *PIM1* expression were inhibited, with *PIM1* change potentially facilitating the transition of iPSC cells from a proliferative stem state to mature cell differentiation [42]. Overall, the interaction between dark genes and their first-order DEG neighbors may uncover crucial signaling mechanisms in the JAK-STAT pathway. Besides, our Cellchat analysis revealed that *EFNA1* and *EPHA1*, as receptor–ligand pairs specifically expressed between DE and MH, might play a key role in iPSC differentiation (Fig. 5G) [43].

## Discussion

Identifying the pre-transition or critical state, along with key molecules, is essential for understanding various biological phenomena. For example, the identification of the pre-transition state or fate decision point in the cell differentiation process plays a crucial role in developing patient-specific tissue regeneration therapies and creating models for diseases related to differentiation. However, conventional methods remain limited in effectiveness and robustness when processing high-dimensional data with substantial noise, as is common in single-cell expression data. Most existing computational methods for single-cell data analysis rely on statistical metrics such as mean and variance derived from gene expression levels. In contrast to gene expression in single-cell data, gene association information remains stable across time and conditions [10], providing reliable characterization of biological processes such as critical transitions. In this study, we introduce a novel computational method at the single-cell level, termed CCNE, which aims to construct a cell-specific causal network for each cell and accurately pinpoint critical states for complex biological systems. By applying the CCNE method to both the simulated dataset and 5 single-cell datasets of different biological processes, we successfully identify their corresponding pre-transition states, validating the capability of the proposed method to detect critical signals at the single-cell level.

Our CCNE method presents the following novelty and benefits. Firstly, from the perspective of analyzing dynamic changes, it more effectively captures critical signals compared to existing critical state detection methods. Secondly, it enables the effective grouping of cells across different time points or cell types by distinguishing cellular heterogeneity based on the CCNE matrix. Thirdly, it facilitates the discovery of CCNE-sensitive “dark genes”, which are typically overlooked by traditional differential expression analysis but potentially crucial in fundamental biological processes. Moreover, our CCNE strategy does not rely on models and operates without the necessity for feature selection or training of parameters. However, despite these advantages, the CCNE method has certain limitations. Specifically, it relies on the availability of a PPI network as the background network. Additionally, interpreting the biological significance of each identified critical point in multi-stage biological processes remains challenging and will be the focus of ongoing research.

## Materials and Methods

### Theoretical background

From the viewpoint of dynamical systems, the complex biological process is often considered a time-evolving system, wherein the pre-transition state signifies a sudden qualitative alteration observed at a bifurcation point [44]. Such a dynamical process can be divided into 3 main phases or states (Fig. 1A): a before-transition state with stability and resilience, a highly sensitive pre-transitional state where cell state transition occurs, and another stable after-transition state. Recently, the theoretical framework of DNB [45] has been applied for the quantitative detection of critical point or state transition point in complex biological processes. In particular, a cluster of DNBs with strong correlations and notable fluctuations emerges as the dynamic system approaches its critical state. Actually, the characteristics of DNB imply that dramatic variations in molecular expression and shifts in their causal regulatory strength can effectively signal the critical transition of the system [46]. Thus, by leveraging the dynamic variations in the causal relations among DNBs and their fluctuations in expression level, it is possible to predict the critical signal of a qualitative or drastic shift.

In this study, to determine continuous causality or functional dependency from a causal gene to an effect gene, we define continuous causality through functional continuity between variables based on a scaling law [20,21], which can be employed to infer direct gene regulatory network from single-cell data. Specifically, consider 2 genes $g_{x}$ and $g_{y}\;$withMdata points or cells, represented as $E\left(g_{x}\right)=\left\{E_{1}\left(g_{x}\right),E_{2}\left(g_{x}\right),\cdots ,E_{M}\left(g_{x}\right)\right\}$ and $E\left(g_{y}\right)=$ $\left\{E_{1}\left(g_{y}\right),E_{2}\left(g_{y}\right),\cdots ,E_{M}\left(g_{y}\right)\right\}$. To quantify the causality or continuity of $g_{y}$ = $f\;\left(g_{x}\right)$ from $g_{x}$ to $g_{y}$ in cell l, a new causal criterion or cross-map mutual information is designed and formulated as follows:

$$w^{\left(l\right)}\left(g_{x},g_{y}\right)g_{y}=pr^{\left(l\right)}\left(f\left(g_{x}\right),g_{y}\right)\text{log}\frac{pr^{\left(l\right)}\left(f\left(g_{x}\right),g_{y}\right)}{pr^{\left(l\right)}\left(g_{x}\right)∙pr^{\left(l\right)}\left(g_{y}\right)}\text{,}$$ (1)

$$\begin{array}{c} {\mathrm{pr}}^{\left(l\right)}\left(f\left(g_{x}\right),g_{y}\right)=\frac{n\left(f\left(E_{\left(l\right)}^{\left(\mathrm{NN}\right)}\left(g_{x}\right)\right),E_{\left(l\right)}^{\left(\mathrm{NN}\right)}\left(g_{y}\right)\right)}{M},\\ {\mathrm{pr}}^{\left(l\right)}\left(g_{x}\right)=\frac{n\left(E_{\left(l\right)}^{\left(\mathrm{NN}\right)}\left(g_{x}\right)\right)}{M},{\mathrm{pr}}^{\left(l\right)}\left(g_{y}\right)=\frac{n\left(E_{\left(l\right)}^{\left(\mathrm{NN}\right)}\left(g_{y}\right)\right)}{M}. \end{array}$$ (2)

Here, $n\left(E_{\left(l\right)}^{\left(\mathrm{NN}\right)}\left(g_{x}\right)\right)$ and $n\left(E_{\left(l\right)}^{\left(\mathrm{NN}\right)}\left(g_{y}\right)\right)$ are the counts of the nearest neighbors/cells of $\;E_{l}\left(g_{x}\right)$ and $\;E_{l}\left(g_{y}\right)\;$ in cell l, respectively, among all M cells. The term $f\left(E_{\left(l\right)}^{\left(\mathrm{NN}\right)}\left(g_{x}\right)\right)\;$ represents the cross-mapped values of $E_{\left(l\right)}^{\left(\mathrm{NN}\right)}\left(g_{x}\right)$ to $E\left(g_{y}\right)$, which are the corresponding points of $E_{\left(l\right)}^{\left(\mathrm{NN}\right)}\left(g_{y}\right)$ in $E\left(g_{y}\right)$. The number of points in the intersection of $n(f\left(E_{\left(l\right)}^{\left(\mathrm{NN}\right)}\left(g_{x}\right)\right))\;\text{and}$ $n\left(E_{\left(l\right)}^{\left(\mathrm{NN}\right)}\left(g_{y}\right)\right)$ is denoted as $n\left(f\left(E_{\left(l\right)}^{\left(\mathrm{NN}\right)}\left(g_{x}\right)\right),E_{\left(l\right)}^{\left(\mathrm{NN}\right)}\left(g_{y}\right)\right)$. A positive statistical dependency value signifies the presence of $g_{x}$’s influence on $g_{y}$ or functional dependency from $g_{x}$ to effect $g_{y}$, implying the existence of a directed edge between them. Therefore, Eq. 1 can be utilized to infer a causal relationship from$\;g_{x}$ to $g_{y}$ and construct the cell-specific causal networks at the single-cell level.

### CCNE tailored to identify the pre-transition state of complex biological process

Based on time-series single-cell data, the quantitative CCNE method is utilized to uncover cell state transitions or the pre-transition state during complex biological process, with the following specific implementation steps outlined.

Step 1: Constructing a cell-specific causal network for each cell. Based on the PPI network, the cell-specific causal network $N^{\left(l\right)}$ for cell $C_{l}$ can be constructed using the causal criterion $w^{\left(l\right)}\;$as defined in Eq. 1. Specifically, for a local network centered on a gene $g^{k}$ within the PPI network, whose first-order neighbors are $g_{j}^{k}$ (j=1,2,⋯,U), if $w^{\left(l\right)}\left(g^{k},g_{j}^{k}\right)$ is positive, it indicates a directed edge $\left(g^{k},g_{j}^{k}\right)$ from gene $g^{k}$ to $g_{j}^{k}$; otherwise, no such directed edge exists (Fig. 1B). By evaluating each neighboring gene $g_{j}^{k}$ in the local network of $\;g^{k}\;$ based on the causal criterion $w^{\left(l\right)}\left(g^{k},g_{j}^{k}\right)$, we can derive the causal subnetwork centered on $g^{k}$. Therefore, this process enables us to infer the cell-specific causal network $N^{\left(l\right)}$ for cell $C_{l}$.

Step 2: Isolating each localized causal network from the cell-specific causal network. Specifically, for a given cell $\;C_{l}$, its cell-specific causal network $\;N^{\left(l\right)}\;$ consists of Vlocalized causal network $\;{\mathrm{LN}}_{k}^{\left(l\right)}\;\left(k=1,2,3,\ldots ,V\right)$, each of which is uniquely identified by a specific gene and connected to its immediate outgoing neighbors (i.e., one gene for one localized network). Thus, each localized causal $\;{\mathrm{LN}}_{k}^{\left(l\right)}$ is represented as a directed subnetwork centered on the specific gene $\;g^{k}$, where the central node $g^{k}$ is connected to Q first-order outgoing neighbors or targets $\;\left\{g_{1}^{k},g_{2}^{k},\ldots ,g_{Q}^{k}\right\}$ via Qout-degree edges.

Step 3: Calculating the local CCNE for each localized causal network. In particular, for the localized causal network $\;{\mathrm{LN}}_{k}^{\left(l\right)}\;$centered on the gene $g^{k}\;$with Q first-order outgoing neighbors, its local CCNE $LH_{k}^{\left(l\right)}$ is conducted as follows:

$$\begin{array}{c} LH_{k}^{\left(l\right)}=-\left[\;\frac{1}{Q}\sum_{j=1}^{Q}P^{\left(l\right)}\left(g^{k},g_{j}^{k}\right)\text{log}P^{\left(l\right)}\left(g^{k},g_{j}^{k}\right)\right]{\mathrm{SD}}^{\left(l\right)}\left(g^{k}\right), \end{array}$$ (3)

with

$$\begin{array}{c} P^{\left(l\right)}\left(g^{k},g_{j}^{k}\right)=\frac{w^{\left(l\right)}\left(g^{k},g_{j}^{k}\right)}{\sum_{i=1}^{Q}w^{\left(l\right)}\left(g^{k},g_{i}^{k}\right)},\\ {\mathrm{SD}}^{\left(l\right)}\left(g^{k}\right)=|\mathrm{SD}\left(E_{g^{k}}\right)-\mathrm{SD}\left({\hat{E}}_{g^{k}}\right)|, \end{array}$$ (4)

where $w^{\left(l\right)}\left(g^{k},g_{j}^{k}\right),$as defined in Eq. 1, signifies the weight between the central gene $g_{k}$ and its jth first-order outgoing neighbor$\;g_{j}^{k}$ . The vectors $E_{g^{k}}$ and ${\hat{E}}_{g^{k}}\;$are represented as follows: $E_{g^{k}}=(E_{g^{k}}^{1},E_{g^{k}}^{2},\cdots ,E_{g^{k}}^{l-1},E_{g^{k}}^{l},E_{g^{k}}^{l+1},\cdots ,E_{g^{k}}^{M})$ and ${\hat{E}}_{g^{k}}=$ $\begin{array}{c} \left(E_{g^{k}}^{1},E_{g^{k}}^{2},\cdots ,E_{g^{k}}^{l-1},E_{g^{k}}^{l+1},\cdots ,E_{g^{k}}^{M}\right) \end{array}$**,** where the symbol $\;E_{g^{k}}^{l}$ denotes the expression of the gene $g^{k}$ in cell $C_{l}$. ${\mathrm{SD}}^{\left(l\right)}\;$can be viewed as evaluating the expression variation of the gene $g^{k}$ in cell $C_{l}$ against other cells.

Step 4: Calculating the cell-specific CCNE for each cell. Specifically, for cell $C_{l}$, the cell-specific CCNE $H^{\left(l\right)}$ is calculated by a cluster of genes with the highest local CCNE, according to the following formula:

$$H^{\left(l\right)}=\sum_{k=1}^{S}{\mathrm{LH}}_{k}^{\left(l\right)},$$ (5)

where the constant S corresponds to the count of genes ranked in the top 5% for the highest local CCNE. Our analysis indicates that parameter S within a certain range does not affect the overall trend of the signal curve (Fig. S7). Furthermore, the average cell-specific CCNE $H_{t}$ at specific time point t, as described in Eq. 6, serves to capture the warning signal of cell state transition.

$$H_{t}=\frac{1}{M}\sum_{l=1}^{M}\;H^{\left(l\right)}.$$ (6)

Close to the pre-transition state or critical state, signaling molecules known as DNBs show the collective behaviors of fluctuations, characterized by a notable increase in molecule expression variation and a rapid strengthening effect of their causal strength. Analyzing the dynamic change of this group of DNB variables at a network level enables the prediction of qualitative state transitions. Consequently, the CCNE $H_{t}$ exhibits a marked increase as the system nears the pre-transition state.

Step 5: Identifying the pre-transition state through the one-sample *t* test. To evaluate the effectiveness of our proposed CCNE $H_{t}$ in detecting critical signals, we employ a one-sample *t* test [47] to ascertain the statistical significance of differences between the pre-transition state and the before-transition state. The formula for the one-sample *t* test statistic SC can be found below as Eq. 7 and is utilized to determine the significance of the deviation of the constant x from the average of the m-dimensional vector $X=\left(x_{1},x_{2},\cdots ,x_{m}\right)$.

$$\mathrm{SC}=\sqrt{m}\frac{\text{mean}\left(X\right)-x}{\mathrm{SD}\left(X\right)}$$ (7)

where the terms $\text{mean}\left(X\right)$ and $\mathrm{SD}\left(X\right)$ refer to the average and standard deviation of vector X, respectively. The SC statistic yields a *P* value that evaluates the significance of statistics between $\text{mean}\left(X\right)$ and constant x. If the *P* value is less than 0.05 (*P* value <0.05), it denotes a statistically significant difference; otherwise, it suggests an absence of significant difference. Therefore, a pre-transition state is reached when the CCNE $H_{t}\;$meets both of the following criteria: (a) $H_{t}\;$is larger than $H_{t-1}$, and (b) $H_{t}$ varies from prior values in a statistically significant manner (*P* value <0.05), as detailed in Section H of the Supplementary Materials.

### Sources of data and functional analysis

To highlight the capabilities of the CCNE method, it has been utilized in both a numerical simulation and the analysis of 5 real-world single-cell datasets: pericyte-to-neuron transition (ID: GSE113036), MEF-to-neuron transition (ID: GSE67310), MHC-to-HCC transition (ID: GSE90047), non-time-series EPCD transition (ID: GSE161277), and iPSC-to-MH transition (ID: GSE81252). Comprehensive descriptions of these datasets are available in Section I of the Supplementary Materials. An analysis of functional enrichment was conducted using the Metascape online tool [48] and the ClusterProfiler package [49]. Pathway-related information is accessible through KEGG.

## Data Availability

The data for pericyte-to-neuron transition (ID: GSE113036), MEF-to-neuron transition (ID: GSE67310), MHC-to-HCC transition (ID: GSE90047), EPCD transition (ID: GSE161277), and iPSC-to-MH transition (ID: GSE81252) were obtained from the NCBI Gene Expression Omnibus (GEO) database at http://www.ncbi.nlm.nih.gov/geo. The source code of the algorithm and related data can be found at https://github.com/zhongjiayuan-fs/CCNE_projects.
