# Supplementary material for: Identification of Critical States in Complex Biological Systems Using Cell-Specific Causal Network Entropy
Source: Research (Wash D C). 2025 Aug 26;8:0852. doi: 10.34133/research.0852 (PMC12379065; doi:10.34133/research.0852)
Supplement: Supplementary 1 — Supplementary Text Figs. S1 to S7 Table S1 [file research.0852.f1.pdf]

Supplementary Information: Identification of critical states in complex biological systems using cell-specific causal network entropy (CCNE)

Contents

A. Describing a dynamic system for numerical modeling S2

B. Constructing a pseudo-temporal trajectory for EPCD data S4

C. Analysis of CCNE and gene expression for critical transition detection S4

D. Clustering of cells based on t-SNE and UMAP S5

E. Dynamic changes of regulatory networks consisting of signaling genes S6

F. Discovering CCNE-sensitive "dark genes" S7

G. Signal curve under varying parameter S S9

H. Describing the identification of the critical state S10

I. Describing the five single-cell datasets of different biological processes S11

J. Runtime evaluations of CCNE under different real-world datasets S12

A. Describing a dynamic system for numerical modeling

Using an 8-node regulatory network model, we analyzed the effectiveness of our CCNE method. Such a regulatory network based on Michaelis-Menten form or Hill bifurcation are commonly used to model gene activity in biological systems, including transcription and translation [1,2]. A set of differential equations can be formulated to represent this 8-node regulatory network, as shown below.

$$\begin{cases} \frac{\mathrm{d}z_1(t)}{dt} = \frac{(4-3|s|)z_2(t)}{10(1+z_2(t))} - \frac{(4+3|s|)}{10} z_1(t) + \zeta_1(t) \\ \frac{\mathrm{d}z_2(t)}{dt} = \frac{(4-3|s|)z_1(t)}{10(1+z_1(t))} - \frac{(4+3|s|)z_2(t)}{10(1+z_2(t))} + \zeta_2(t) \\ \frac{\mathrm{d}z_3(t)}{dt} = \frac{(6|s|-10)}{10} + \frac{(5-3|s|)}{10(1+z_1(t))} + \frac{(5-3|s|)}{10(1+z_2(t))} - z_3(t) + \zeta_3(t) \\ \frac{\mathrm{d}z_4(t)}{dt} = \frac{(6|s|-12)}{10} + \frac{(6-3|s|)z_1(t)}{10(1+z_1(t))} + \frac{(6-3|s|)z_2(t)}{10(1+z_2(t))} - \frac{6}{5} z_4(t) + \zeta_4(t) \\ \frac{\mathrm{d}z_5(t)}{dt} = \frac{(6|s|-14)}{10} + \frac{(7-3|s|)z_1(t)}{10(1+z_1(t))} + \frac{(7-3|s|)z_2(t)}{10(1+z_2(t))} - \frac{7}{5} z_5(t) + \zeta_5(t) \\ \frac{\mathrm{d}z_6(t)}{dt} = -\frac{3}{5} + \frac{1}{10(1+z_1(t))} + \frac{1}{10(1+z_2(t))} + \frac{1}{5(1+z_5(t))} + \frac{1}{5(1+z_7(t))} \\ + \frac{z_8(t)}{5(1+z_8(t))} - \frac{8}{5} z_6(t) + \zeta_6(t) \\ \frac{\mathrm{d}z_7(t)}{dt} = \frac{z_8(t)}{10(1+z_8(t))} - \frac{19}{10} z_7(t) + \zeta_7(t) \\ \frac{\mathrm{d}z_8(t)}{dt} = \frac{z_7(t)}{10(1+z_7(t))} - \frac{19}{10} z_8(t) + \zeta_8(t) \end{cases} \quad (S1)$$

Here, s is a scalar control parameter and $\varsigma_i(t)$ (i = 1, 2, ···, 8) represents Gaussian noises with zero means and covariances given by $k_{ij} = Cov(\varsigma_i, \varsigma_j)$. The concentrations of mRNA-i are represented by $z_i(t)$ (i=1,2···,8). According to Eq.(S1), the degradation rates of mRNAs can be described as the vector $\mathrm{R}=(\frac{(4+3|s|)}{10}, \frac{(4+3|s|)}{10}, 1, \frac{6}{5}, \frac{7}{5}, \frac{8}{5}, \frac{19}{10}, \frac{19}{10})$. $\overline{Z} = (\overline{z_1}, \overline{z_2}, \cdots, \overline{z_8}) = (0, 0, \cdots, 0)$ is denoted as the stable equilibrium point of the dynamic system described by Eq. (S1). By applying the Euler scheme, Equation (S1) can be discretized into the following set of discrete equations using a small time interval of Δt .

$$\begin{cases} z_1(k+1)=z_1(k)+\left[\dfrac{(4-3|s|)z_2(k)}{10(1+z_2(k))}-\dfrac{(4+3|s|)}{10}z_1(k)+\zeta_1(k)\right]\Delta t \\ z_2(k+1)=z_2(k)+\left[\dfrac{(4-3|s|)z_1(k)}{10(1+z_1(k))}-\dfrac{(4+3|s|)z_2(k)}{10(1+z_2(k))}+\zeta_2(k)\right]\Delta t \\ z_3(k+1)=z_3(k)+\left[\dfrac{(6|s|-10)}{10}+\dfrac{(5-3|s|)}{10(1+z_1(k))}+\dfrac{(5-3|s|)}{10(1+z_2(k))}-z_3(k)+\zeta_3(k)\right]\Delta t \\ z_4(k+1)=z_4(k)+\left[\dfrac{(6|s|-12)}{10}+\dfrac{(6-3|s|)z_1(k)}{10(1+z_1(k))}+\dfrac{(6-3|s|)z_2(k)}{10(1+z_2(k))}-\dfrac{6}{5}z_4(k)+\zeta_4(k)\right]\Delta t \\ z_5(k+1)=z_5(k)+\left[\dfrac{(6|s|-14)}{10}+\dfrac{(7-3|s|)z_1(k)}{10(1+z_1(k))}+\dfrac{(7-3|s|)z_2(k)}{10(1+z_2(k))}-\dfrac{7}{5}z_5(k)+\zeta_5(k)\right]\Delta t \\ z_6(k+1)=z_6(k)+\left[-\dfrac{3}{5}+\dfrac{1}{10(1+z_1(k))}+\dfrac{1}{10(1+z_2(k))}+\dfrac{1}{5(1+z_5(k))}+\dfrac{1}{5(1+z_7(k))}\right. \\ \left.+\dfrac{z_8(k)}{5(1+z_8(k))}-\dfrac{8}{5}z_6(k)+\zeta_6(k)\right]\Delta t \\ z_7(k+1)=z_7(k)+\left[\dfrac{z_8(k)}{10(1+z_8(k))}-\dfrac{19}{10}z_7(k)+\zeta_7(k)\right]\Delta t \\ z_8(k+1)=z_8(k)+\left[\dfrac{z_7(k)}{10(1+z_7(k))}-\dfrac{19}{10}z_8(k)+\zeta_8(k)\right]\Delta t \end{cases} \qquad (S2)$$

Where $Z(k)$ represents the vector $Z(t)$ at the time instant $k\Delta t$. The Jacobian matrix of Eq. (S2) is expressed as $J=\left.\dfrac{\partial f(Z(k);S)}{\partial Z}\right|_{Z=\bar{Z}}$, with

$$J=e^{\Delta t \cdot A} \qquad (S3)$$

By taking $\Delta t=1$, it is feasible to derive eight distinct eigenvalues from Eq. (S3). The primary eigenvalue satisfies the criterion $0.66^{|s|}\rightarrow 1$ when $s\rightarrow 0$, indicating that the principal eigenvalue of the system described by Equation (S1) asymptotically approaches 0 from the negative domain as $s\rightarrow 0$. As a result, the equilibrium point is deemed stable when $s\in(0,1]$. This specific parameter $s=0$ denotes the bifurcation point, signifying a qualitative shift in the system. Specifically, when $s<0$, real parts of all the eigenvalues for the Jacobian matrix of Eq. (S1) at the equilibrium are negative, which means that originally the equilibrium point $(\bar{z}_1,\bar{z}_2,\cdots,\bar{z}_8)=(0,0,\cdots,0)$ is stable. When the parameter s approaches 0, the largest real part among the all eigenvalues approaches 0 and then becomes positive after passing $s=0$, which means that the original stable equilibrium $(\bar{z}_1,\bar{z}_2,\cdots,\bar{z}_8)$ becomes unstable and further bifurcates into a qualitatively different periodic solution (actually, a Hopf bifurcation occurs). Utilizing the theoretical model outlined in Equation (S2), numerical simulation datasets were generated for the 8-node network by systematically varying the parameter within the range of -0.5 to 0.2.

B. Constructing a pseudo-temporal trajectory for EPCD data

The EPCD data pertaining to epithelial cell deterioration in colorectal cancer [3] were processed using the Seurat pipeline. To account for inter-patient biological variability, batch effects were removed using the Harmony R package. We employed a pseudo-time inference strategy using the Monocle algorithm to reconstruct the trajectory of EPCD progression. Clustering was performed using the FindClusters function with a resolution of 1.0 for all cell types and 0.6 for epithelial subpopulations. Specifically, the pseudo-temporal trajectory of EPCD was reconstructed based on three major epithelial subpopulations: benign cells, TUBA1B+H2AFZ+HMGB2+HIST1H4C+ transitional cells, and malignant cells (Figure S1A), which were further categorized into six distinct clusters: Cluster 1 (950 cells), Cluster 2 (450 cells), Cluster 3 (650 cells), Cluster 4 (450 cells), Cluster 5 (450 cells), and Cluster 6 (738 cells) (Figure S1B). Therefore, for non-time-series single-cell dataset of EPCD, the progression of EPCD can be categorized into six distinct clusters by constructing a pseudo-temporal trajectory.

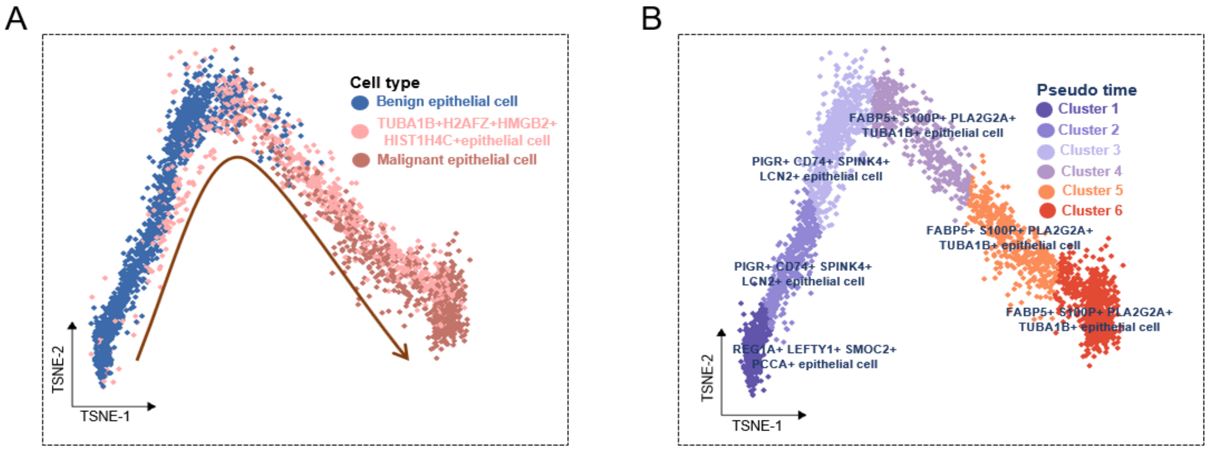

Figure S1: (A) A pseudo-temporal trajectory of epithelial cell deterioration was reconstructed based on three distinct subpopulations. (B) The EPCD progression was further divided into six distinct clusters.

C. Analysis of CCNE and gene expression for critical transition detection

To compare CCNE and gene expression for critical transition detection, we analyzed the dynamic changes and identified critical states of complex biological processes from the perspective of gene expression patterns. Specifically, similar to the CCNE-based approach, for each cell, the average expression of the highest 5% of genes ranked by expression value was employed to analyze the dynamic changes. It is seen from Figure S2 that gene expression does well not characterize the dynamic changes and identifies critical states of complex biological processes as effectively as CCNE value.

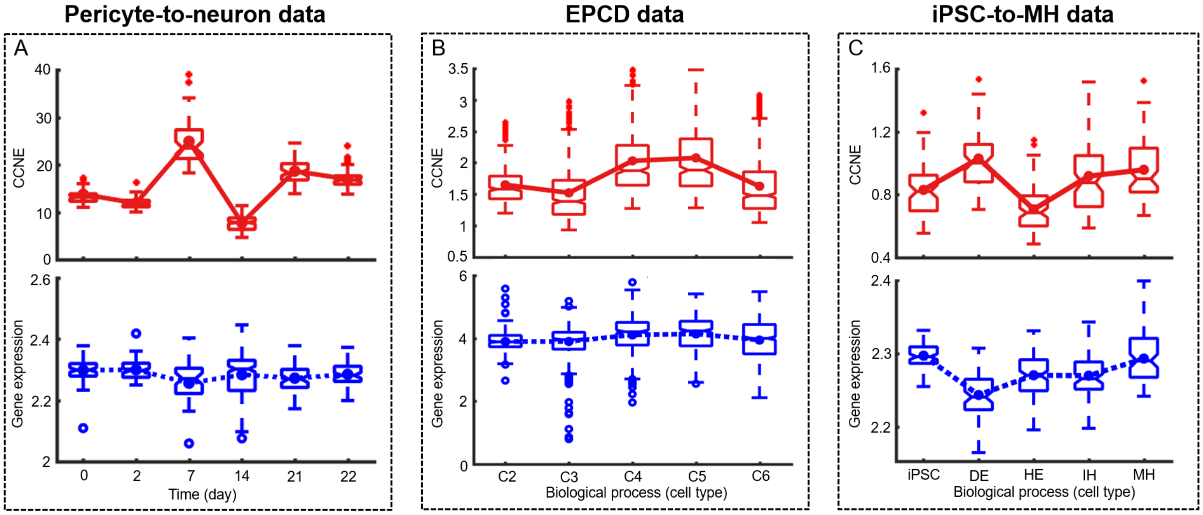

Figure S2: Comparative performance of CCNE and gene expression in capturing critical transitions for (A) pericyte-to-neuron data, (B) EPCD data, and (C) iPSC-to-MH data. For each subfigure, red curve indicates CCNE scores, while blue curve represents gene expression levels.

D. Clustering of cells based on t-SNE and UMAP

In addition to identification of the pre-transition state, our approach can transform gene expression data into an CCNE matrix, enabling CCNE-based cell clustering analysis. In order to comprehensively evaluate the robustness of CCNE-based clustering, we performed uniform manifold approximation and projection (UMAP) and t-distributed stochastic neighbor embedding (t-SNE) analysis to evaluate CCNE-based clustering across different time points. As shown in Figure S3, the UMAP results similarly distinguish cellular states at various time points as effectively as the t-SNE analysis, indicating that the day-to-day transitions can also be captured by CCNE scores using UMAP-based clustering.

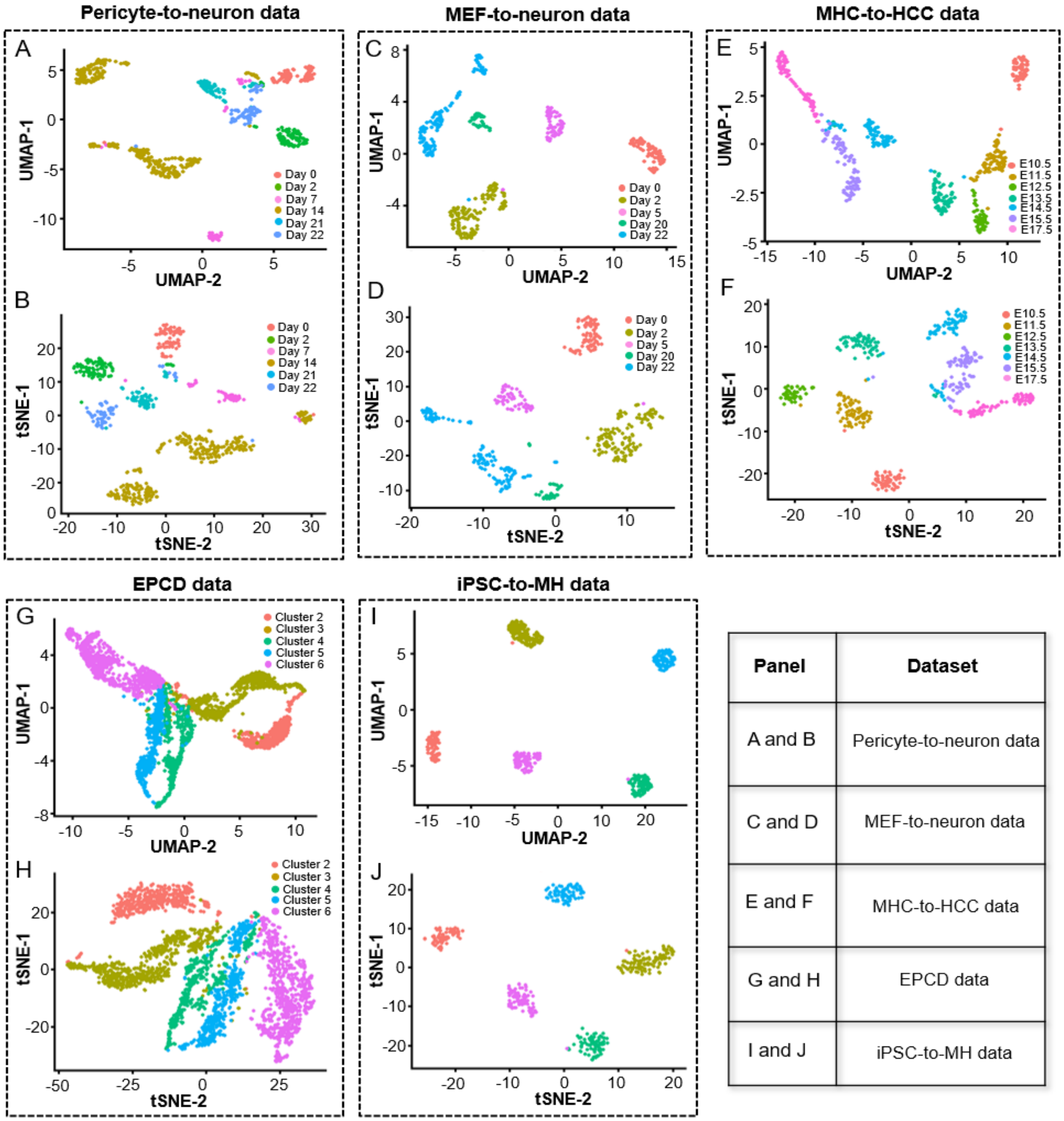

Panel	Dataset
A and B	Pericyte-to-neuron data
C and D	MEF-to-neuron data
E and F	MHC-to-HCC data
G and H	EPCD data
I and J	iPSC-to-MH data

Figure S3: Comparison of clustering analysis using UMAP and TSNE. UMAP and t-SNE were applied to visualize CCNE-based clustering for (A)-(B) pericyte-to-neuron data, (C)-(D) MEF-to-neuron data, (E)-(F) MHC-to-HCC data, (G)-(H) EPCD data, and (I)-(J) iPSC-to-MH data.

E. Dynamic changes of regulatory networks consisting of signaling genes

At the identified pre-transition states, we selected the top 5% of genes with the highest local CCNE as signaling genes. These genes were integrated into the protein-protein interaction (PPI) network, from which the most extensive connected subgraph was extracted to analyze the dynamic evolution of the regulatory network for signaling genes. In the pericyte-to-neuron data, the evolution of the regulatory network across all six time points is illustrated in Figure S4A. Notably, a distinct alteration in the network structure at day 7 was observed, signaling an early onset of differentiation into induced neurons after

day 7 [4]. For the MEF-to-neuron data, a marked change in the network was detected on day 20 (Figure S4B), suggesting the differentiation of mouse embryonic fibroblasts into induced neurons at day 22 [5].

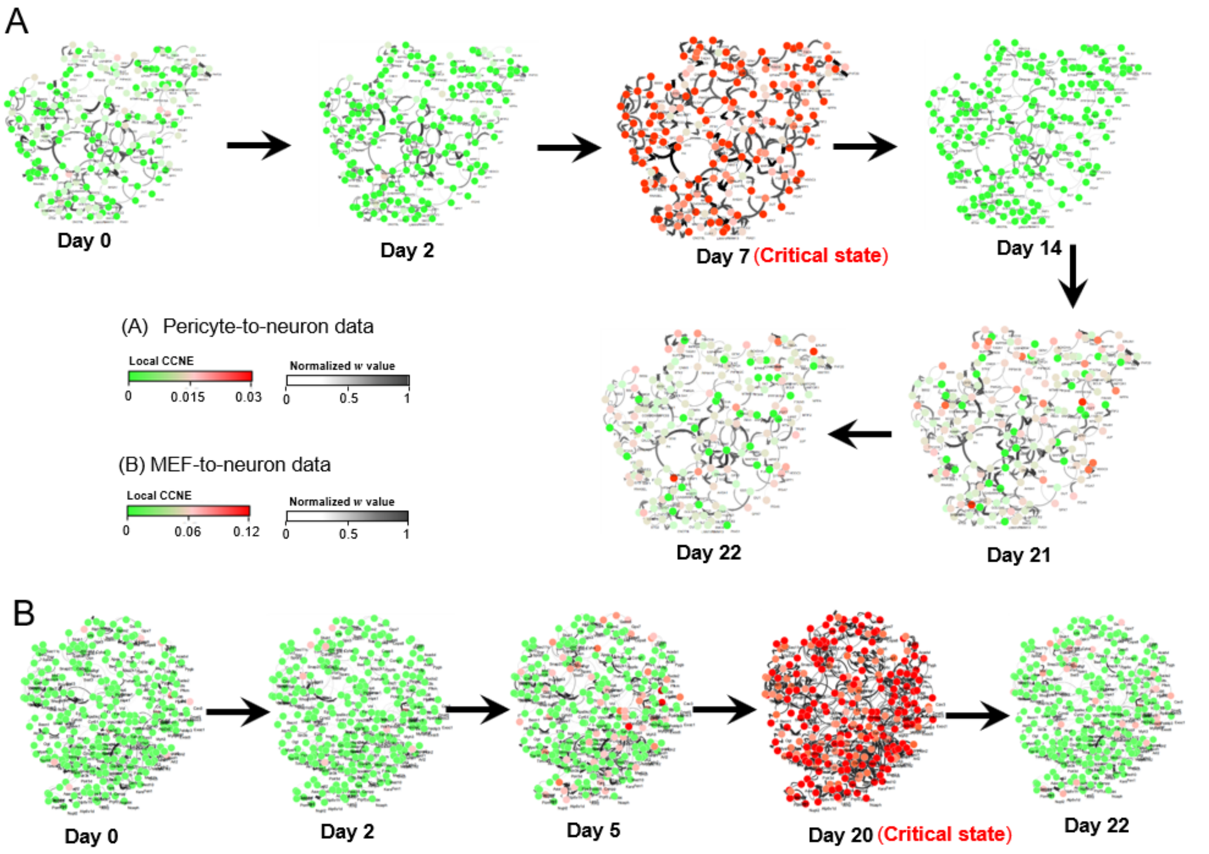

Figure S4: (A) Temporal evolution of the regulatory network in the pericyte-to-neuron data. (B) The dynamic evolution of the regulatory network for the MEF-to-neuron data.

F. Discovering CCNE-sensitive "dark genes"

In our research, some genes within the category of DNBs or trigger molecules demonstrate no variance in expression at the molecular level, yet display significant responsiveness to fluctuations in the CCNE score. These genes are classified as "dark genes" based on two criteria: (i) they exhibit no significant differences in gene expression levels, and (ii) they display a notable differentiation between pre-transition and before-transition states in terms of CCNE score. Specifically，we conducted a comparative analysis of the dynamic changes in gene expression and CCNE scores of trigger molecules, (top 5% genes with the highest local CCNE score) to discover "dark genes". The 'dark genes' for the three different datasets: pericyte-to-neuron, MEF-to-neuron, and iPSC-to-MH data can be found at https://github.com/zhongjiayuan/scCNE_project. These genes exhibit a notable difference between the critical point and non-critical point at the CCNE level, but this distinction is not evident at the gene expression level.

Moreover, we performed Gene Ontology (GO) enrichment and pathway annotation analyses of the identified “dark genes” to elucidate their functional relevance in developmental processes. For pericyte-

to-neuron data, “dark genes” showed significant enrichment in biological pathways including endoderm formation, regulation of nervous system development, and protein processing/maturation (Figure S5A). These pathways are functionally associated with cellular proliferation, tissue differentiation, and morphogenesis during embryonic development. Notably, the dark genes *MMP2* and *ITGA7* (shown in Table 2 of the main text) were specifically enriched in the endoderm formation pathway, suggesting a direct role in early embryonic tissue differentiation. When applied to MEF-to-neuron data, as presented in Figure S5B, “dark genes” were predominantly enriched in Wnt signaling pathway, cellular amino acid metabolic process, regulation of cell growth and protein import. These pathways are known to play pivotal roles in embryonic development by modulating cell fate specification, epigenetic regulation, and metabolic support. Specifically, dark gene *Ripk1* (listed in Table 2 of the main text) was enriched in pathways associated with cellular proliferation and organ morphogenesis, further supporting the developmental significance of dark genes.

In addition, some "dark genes" can regulate various non-coding RNAs [6] (Figures S6A-B). Specifically, *ITGA7, MMP2,* and *LAMTOR1* are likely to interact with specific microRNAs (miRNAs) that are significantly enriched in embryogenesis-related pathways (Figure S6C), such as the Wnt signaling pathway [7], the JAK-STAT signaling pathway [8], and the FoxO signaling pathway [9], suggesting their potential roles as regulatory factors during embryonic development. Similarly, "dark genes" including *Ccnd2, Dctn2, Fars2,* and *Ripk1* may contribute to embryonic development through the regulation of miRNAs associated with key developmental pathways (Figure S6D).

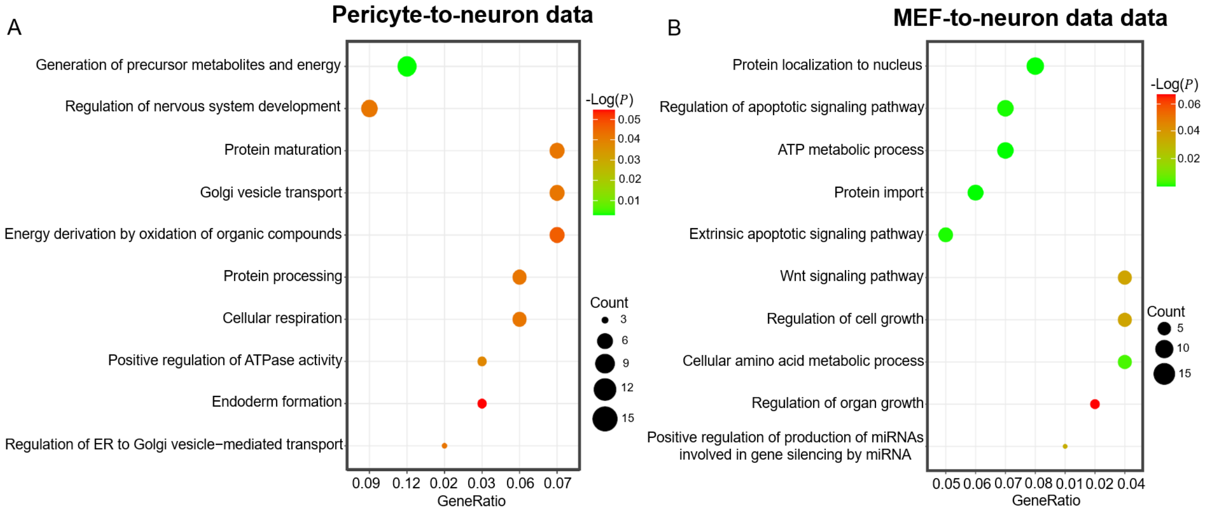

Figure S5: Results of GO enrichment analysis for dark genes identified in (A) pericyte-to-neuron data and (B) MEF-to-neuron data. The analysis highlights that "dark genes" are enriched in biological processes associated with embryonic development.

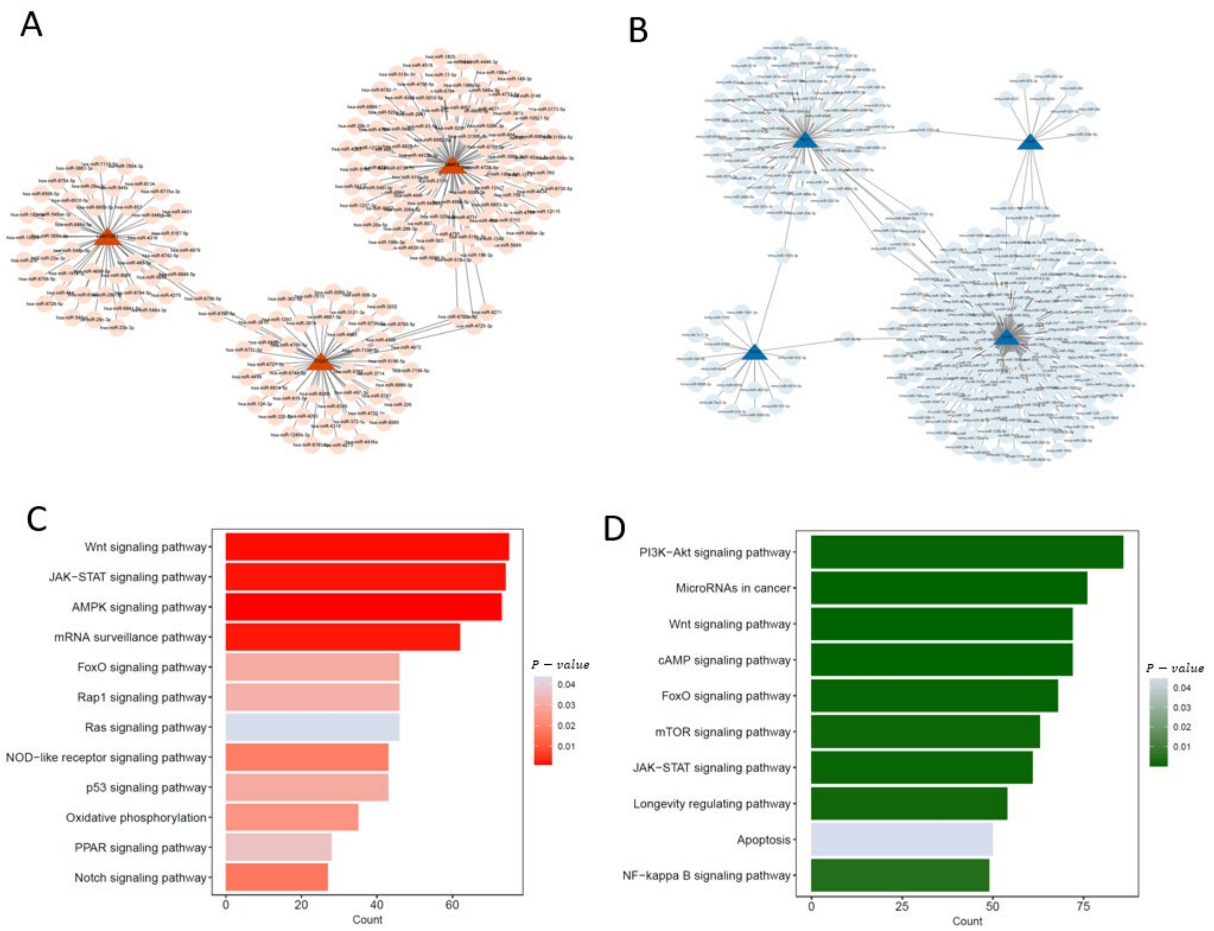

Figure S6: (A) In pericyte-to-neuron data, "dark genes" such as *ITGA7*, *MMP2*, and *LAMTOR1* are capable of regulating various microRNAs. (B) In MEF-to-neuron data, "dark genes" including *Ccnd2, Dctn2, Fars2, and Ripk1* regulate various microRNAs. (C) In pericyte-to-neuron data, miRNAs regulated by *ITGA7, MMP2,* and *LAMTOR1* are significantly enriched in embryogenesis-related signaling pathways. (D) In MEF-to-neuron data, miRNAs regulated by *Ccnd2, Dctn2, Fars2,* and *Ripk1* show significant enrichment in pathways associated with embryonic development.

G. Signal curve under varying parameter S

We analyzed the pericyte-to-neuron and MEF-to-neuron datasets using different values of the parameter S, ranging from the top 3% to 10%, to assess critical signals. As illustrated in Figure S7, the CCNE index consistently identifies the critical point with a similar trend. These results indicate that parameter S within this range (typically from the top 3% to 10%) do not alter the overall trend of the signal curve, demonstrating the robustness of CCNE against the variation of parameter S.

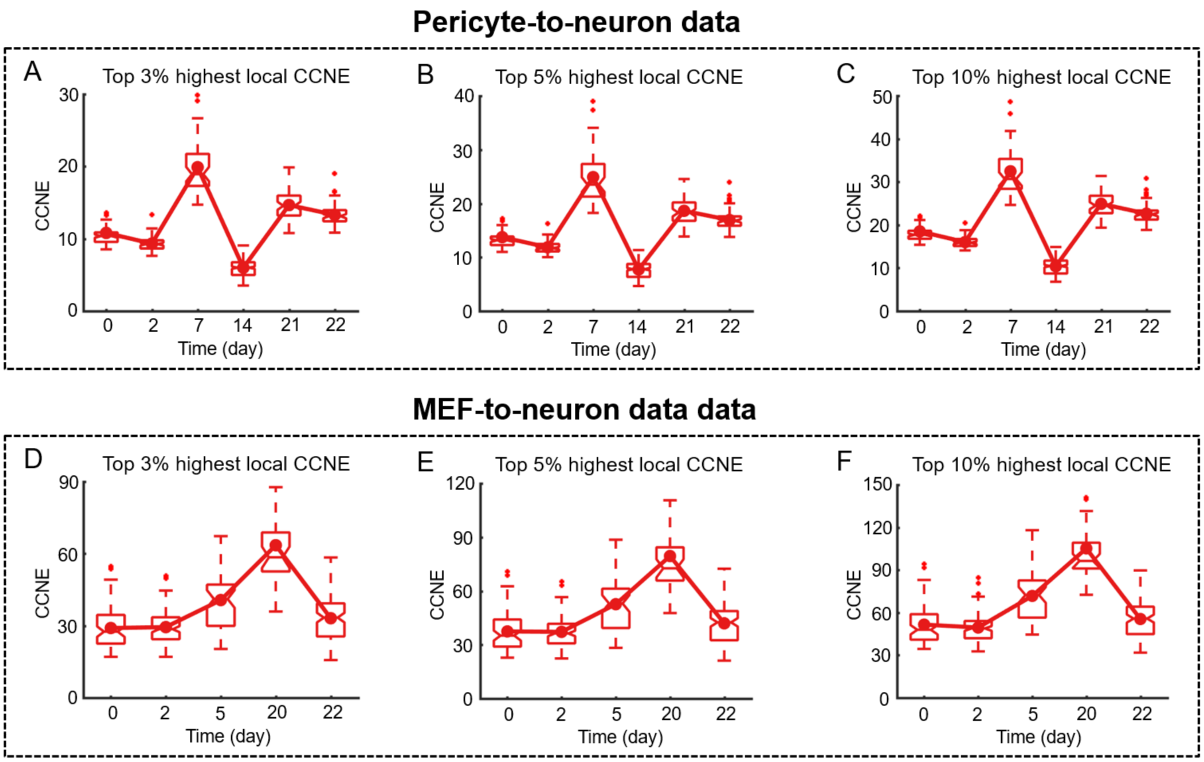

Figure S7: the critical signals observed in the pericyte-to-neuron and MEF-to-neuron datasets with different settings of the adjustable parameter S. In the case of the pericyte-to-neuron data, S is set as (A) the number of top 3% genes with highest local CCNE, (B) the number of top 5% genes with highest local CCNE, and (C) the number of top 10% genes with highest local CCNE, respectively. Similarly, for the MEF-to-neuron data, S is set as (D) the number of top 3% genes with highest local CCNE, (E) the number of top 5% genes with highest local CCNE, and (F) the number of top 10% genes with highest local CCNE, respectively.

H. Describing the identification of the critical state

To assess the efficacy of the CCNE score in quantifying critical behavior, we utilize a one-sample t-test to ascertain the presence of a statistically significant distinction between the before-transition and pre-transition states. The formula for the one-sample t-test statistic SC can be found below Eq. (S1) and is utilized to determine the significance of the deviation of the constant x from the mean of the m-dimensional vector $\boldsymbol{X} = (x_1, x_2, \cdots, x_m)$.

$$SC = \sqrt{m}\frac{\mathrm{mean}(\boldsymbol{X}) - x}{\mathrm{SD}(\boldsymbol{X})} \quad \text{(S4)}$$

The term $\mathrm{mean}(\boldsymbol{X})$ refers to the average value of vector $\boldsymbol{X}$, while $\mathrm{SD}(\boldsymbol{X})$ indicates its standard deviation. Statistic SC yields a p-value that assesses the significance of differences in statistics between the $\mathrm{mean}(\boldsymbol{X})$ and the constant x. A statistically significant difference is observed between the $\mathrm{mean}(\boldsymbol{X})$ and x when the p-value is less than 0.05 (p<0.05). In the context of our study, a time point $t > 2$ is regarded as a pre-transition state if CCNE H_t meets both of the following criteria: (i) H_t is greater than H_{t-1}, and (ii) H_t demonstrates a statistically significant difference (p<0.05) from the average of the vector H=$(H_1, H_2, \cdots, H_{t-1})$. When the following condition is satisfied by the CCNE H_2: (i) H_2 is greater than H_1

and (ii) H_2 indicates a statistically significant difference (p<0.05) from the average of the vector H=(H_1, H_3), the time point $t = 2$ can be interpreted as indicative of the pre-transition state.

I. Describing the five single-cell datasets of different biological processes

The functionality of the CCNE method has been demonstrated through its application to five real-world datasets: pericyte to neuron transition (pericyte-to-neuron data, ID: GSE113036), mouse embryonic fibroblast (MEF) to neuron transition (MEF-to-neuron data, ID: GSE67310), mouse hepatoblast cell (MHC) to hepatocyte and cholangiocyte cell (HCC) transition (MHC-to-HCC data, ID: GSE90047), epithelial cell deterioration transition (EPCD) transition (EPCD data, ID: GSE161277), and induced pluripotent stem cell (iPSC) to mature hepatocytes (MH) transition (IPSC-to-MH, ID: GSE81252) sourced from the Gene Expression Omnibus (GEO) database (http://www.ncbi.nlm.nih.gov/geo/). The detailed descriptions and sources of the datasets are listed below.

The pericyte-to-neuron data [4] originated from the direct reprogramming of adult human brain pericytes into induced neuronal cells. Cell counts were obtained at various time points, including day 0 (76 cells), day 2 (86 cells), day 7 (48 cells), day 14 (283 cells), day 21 (61 cells), and day 22 (69 cells). The normalized data can be accessed through the GEO database under accession number GSE113036.

The MEF-to-neuron data [5] was obtained from a reprogramming process of mouse embryonic fibroblasts (MEF) to induced neuronal cells, which includes a total of 405 single cells representing eleven distinct cell types. The reprogramming of MEF was initiated at day 0, resulting in 73 cells, with additional samples collected at day 2 (128 cells), day 5 (55 cells), day 20 (33 cells), and day 22 (116 cells) post-induction. The normalized data utilized in this analysis were sourced from the GEO database under accession number GSE67310.

The MHC-to-HCC dataset [10] was obtained through the differentiation process of mouse hepatoblast cells (MHC) into hepatocytes and cholangiocytes cells (HCC). This dataset comprises a total of 447 single cells collected at seven time points, including 54 single cells at embryonic day 10.5 (E10.5), 70 at E11.5, 41 at E12.5, 65 at E13.5, 70 at 14.5, 77 at 15.5, and 70 at E17.5. The normalized data can be accessed from the GEO database under the accession number GSE90047.

The progression of epithelial cell deterioration (EPCD) data [3] was classified into six distinct clusters: cluster 1 (950 cells), cluster 2 (450 cells), cluster 3 (650 cells), cluster 4 (45 cells), cluster 5 (450 cells), and cluster 6 (738 cells) based on a pseudo-temporal trajectory. The gene expression profiling data can be acquired from the GEO database under accession number GSE161277.

The iPSC-to-MH data [11] pertaining to the trans-differentiation process from induced pluripotent stem cells (iPSC) to mature hepatocytes (MH) was obtained and classified into five distinct clusters: iPSC (80 cells), definitive endoderm (DE) (70 cells), hepatic endoderm (HE) (113 cells), immature hepatocytes (IH) (81 cells), and mature hepatocytes (MH) (81 cells). The normalized data is accessible through the GEO database under accession number GSE81252.

J. Runtime evaluations of CCNE under different real-world datasets

We conducted empirical runtime evaluations of CCNE on various real-world datasets (Table S1), demonstrating its computational performance across different data scales. To enhance practical usability, we provide a Python implementation that accommodates user preferences and computing environments. In addition, the current Python version supports parallel computing, enabling faster processing of large datasets. These enhancements improve the scalability and applicability of our CCNE for real-world scenarios.

Table S1. Runtime evaluations of CCNE method under different real-world datasets

Dataset	Cell number	Empirical runtime (s)
Pericyte-to-neuron data	603	196.8
MEF-to-neuron data	405	73.6
MHC-to-HCC data	447	51.9
iPSC-to-MH data	425	74.9
EPCD data	3688	1943.3

References

[1] Gardner, T. S., Cantor, C. R. & Collins, J. J. Construction of a genetic toggle switch in Escherichia coli. Nature 403, 339–342 (2000).

[2] O'Brien, E. L., Van Itallie, E. & Bennett, M. R. Modeling synthetic gene oscillators. Math. Biosci. 236, 1–15 (2012).

[3] Huang X, Han C, Zhong J, Hu J, Jin Y, Zhang Q, Luo W, Liu R, Ling F. Low expression of the dynamic network markers FOS/JUN in pre-deteriorated epithelial cells is associated with the progression of colorectal adenoma to carcinoma. J Transl Med. 2023 Jan 25;21(1):45.

[4] Karow M, Camp JG, Falk S, Gerber T et al. Direct pericyte-to-neuron reprogramming via unfolding of a neural stem cell-like program. Nat Neurosci 2018; 21(7):932-940.

[5] Treutlein B, Lee QY, Camp JG, Mall M et al. Dissecting direct reprogramming from fibroblast to neuron using single-cell RNA-seq. Nature 2016; 534(7607):391-5.

[6] Chen Y, Wang X. miRDB: an online database for prediction of functional microRNA targets. Nucleic Acids Res. 2020; 48(D1):D127-D131.

[7] Sengupta S, Nie J, Wagner RJ, Yang C, Stewart R, Thomson JA. MicroRNA 92b controls the G1/S checkpoint gene p57 in human embryonic stem cells. Stem Cells. 2009; 27(7):1524-8.

[8] Omeljaniuk WJ, Laudański P, Miltyk W. The role of miRNA molecules in the miscarriage process. Biol Reprod. 2023; 109(1):29-44.

[9] Xi B, An X, Yue Y, Shen H, Han G, Yang Y, Zhao S. Identification and profiling of microRNAs during sheep's testicular development. Front Vet Sci. 2025; 12:1538990.

[10] Yang L, Wang WH, Qiu WL, Guo Z et al. A single-cell transcriptomic analysis reveals precise pathways and regulatory mechanisms underlying hepatoblast differentiation. Hepatology 2017 Nov;66(5):1387-1401.
[11] Camp JG, Sekine K, Gerber T, Loeffler-Wirth H et al. Multilineage communication regulates human liver bud development from pluripotency. Nature 2017 Jun 22;546(7659):533-538.
